# Relapse surveillance of acute myeloid leukemia patients in first remission after consolidation chemotherapy: diagnostic value of regular bone marrow aspirations

**DOI:** 10.1007/s00277-022-04862-3

**Published:** 2022-05-20

**Authors:** Sebastian E. Koschade, Jan A. Stratmann, Fabian Finkelmeier, Sebastian Wagner, Jörg Chromik, Björn Steffen, Hubert Serve, Christian H. Brandts, Olivier Ballo

**Affiliations:** 1 Department of Medicine, Hematology/Oncology, University Hospital Frankfurt, Goethe University, Theodor-Stern-Kai 7, 60590 Frankfurt am Main, Germany; 2 Department of Medicine, Gastroenterology, Hepatology and Endocrinology, University Hospital Frankfurt, Goethe University, Frankfurt am Main, Germany; 3 University Cancer Center Frankfurt (UCT), University Hospital, Goethe University, Frankfurt am Main, Germany

**Keywords:** AML, Relapse surveillance, Bone marrow aspiration, Peripheral blood smears, CBC

## Abstract

The optimal follow-up care for relapse detection in acute myeloid leukemia (AML) patients in first remission after consolidation therapy with intensive chemotherapy is not established. In this retrospective study, we evaluate the diagnostic value of an intensive relapse surveillance strategy by regular bone marrow aspirations (BMA) in these patients. We identified 86 patients with newly diagnosed non-promyelocytic AML who had reached complete remission (CR) after intensive induction and consolidation chemotherapy between 2007 and 2019. Annual relapse rates were 40%, 17%, and 2% in years 1–3, respectively. Patients in CR were surveilled by BMA scheduled every 3 months for 2 years, followed by BMA every 6 months. This surveillance regimen detected 29 of 55 relapses (53%), 11 of which were molecular relapses (20%). The remaining 26 of 55 relapses (47%) were diagnosed by non-surveillance BMA prompted by specific suspicion of relapse. Most patients showed concurrent morphological abnormalities in peripheral blood (PB) at time of relapse. Seven percent of all morphological relapses occurred without simultaneous PB abnormalities and would have been delayed without surveillance BMA. Intensified monthly PB assessment paired with BMA every 3 months during the first 2 years may be a highly sensitive relapse surveillance strategy.

## Introduction

Acute myeloid leukemia (AML) is an aggressive malignant disease of the hematopoietic system and the most frequent acute leukemia in adults [1]. Curative treatment mainly consists of high-intensity induction chemotherapy and consolidation chemotherapy or primary allogeneic stem cell transplantation [1]. The majority of patients in remission eventually experience disease relapse [2]. Relapsed patients are treated with reinduction therapy followed by secondary allogeneic stem cell transplantation or receive either low-intensity antileukemic therapy or best supportive care [1].

The optimal follow-up strategy for relapse detection in AML patients in first remission after definitive consolidation therapy with intensive chemotherapy is not established. Published protocols suggest surveillance strategies ranging from no post-remission surveillance BMA at all [3, 4] to frequent BMAs every 2 to 3 months [5, 6]. Current guidelines (NCCN [7], ESMO [2], DGHO [8]) generally recommend 3-monthly PB assessment during the first 2–5 years for patients in CR after definitive consolidation chemotherapy. However, there is little evidence for the diagnostic utility of specific relapse monitoring strategies [9]. Additionally, recommendations for source material and sampling frequency vary for combined morphological and molecular relapse surveillance and the optimal surveillance strategy is not established [2, 7, 10]. Thus, relapse monitoring strategies differ between institutions and individual physicians, attempting to balance relapse risk, patients’ choice, the availability of molecular measurable residual disease (MRD) markers, applicable relapse treatment options, and perceived benefits of early relapse detection. Our institution implemented an intensive relapse surveillance policy generally consisting of bone marrow aspiration (BMA) scheduled in addition to peripheral blood (PB) assessment every 3 months for 2 year after completion of consolidation chemotherapy and CR confirmation, followed by BMA every 6 months up to 5 years.

Given the paucity of available data, we conducted a single-center retrospective study and analyzed the diagnostic value of intensive relapse surveillance by regular bone marrow aspiration in addition to peripheral blood assessment in AML patients in first remission after consolidation chemotherapy that were treated at our clinic from 2007 to 2019. Secondary objectives were determination of time to relapse and comparison of peripheral blood and bone marrow morphological evaluation at time of relapse.

## Methods

### Patients

All patients newly diagnosed with AML (excluding APL) according to WHO criteria [11] who had completed intensive induction chemotherapy and consolidation chemotherapy between 2007 and 2019 at our institution, achieved complete remission (CR) [12] after consolidation chemotherapy, and visited at least once for follow-up care were retrospectively included. Remission status was assessed by morphological examination, multicolor flow cytometry (not at MRD detection sensitivity), and, when available, molecular testing of bone marrow aspirate (BMA) for measurable residual disease (MRD) by real-time qPCR for NPM1-mutated AMLs and core binding factor AMLs. Patients with persistent MRD in BMA after consolidation chemotherapy were excluded. The screening period consisted of the period between confirmation of CR by bone marrow aspiration after completion of consolidation chemotherapy and either relapse or last follow-up if no relapse was detected. Study group and screening period were predefined. Standard induction chemotherapy consisted of cytarabine 100 mg/m^2^ given continuously for 7 days combined with daunorubicin 60 mg/m^2^ given for 3 days (7 + 3). Patients under the age of 60 received either a second induction therapy cycle of 7 + 3 if they achieved bone marrow blast clearance on day 15 after start of induction therapy, or they received a salvage induction therapy cycle consisting of cytarabine 3000 mg/m^2^ every 12 h for 3 days and mitoxantrone 10 mg/m^2^ for 3 days (HAM) if blast clearance was not achieved on day 15 [13]. Patients above the age of 60 only received a second induction therapy cycle with HAM (with reduced cytarabine dose of 1000 mg/m^2^) if they did not achieve bone marrow blast clearance on day 15. Consolidation chemotherapy was generally administered to patients younger than 60 years who achieved complete remission as three courses of high-dose cytarabine (3 g/m^2^ intravenously over 3 h per q12 h on days 1–3) not earlier than 1 week after attaining CR (HDAC) [14]. Patients older than 60 years generally received two courses of intermediate-dose cytarabine (1 g/m^2^ intravenously over 3 h per q12 h on 3 days (IDAC)) [10]. A minority of patients were enrolled in clinical trials and received experimental therapy in addition to cytarabine-based consolidation chemotherapy. Allogeneic hematopoietic stem cell transplantation instead of consolidation chemotherapy was recommended for patients with ELN intermediate or adverse risk [14]. Patients with primary allogeneic stem cell transplantation as consolidation therapy were not included. Standard antimicrobial prophylaxis consisted of levofloxacin and posaconazol [15, 16]. Transfusion thresholds were Hb < 8.0 g/dL (2007–08/2014)/Hb ≤ 7.0 g/dL (after 08/2014) and/or platelet count < 10/nL, except for febrile patients (Hb ≤ 8.0 g/dL and/or platelet count < 20/nL). Standard relapse surveillance consisted of BMA scheduled every 3 months for 2 years after completion of consolidation chemotherapy and CR conformation, followed by BMA every 6 months. Patients with suspected relapse due to clinical symptoms and/or peripheral blood abnormalities underwent non-surveillance BMA. Patients with relapse of AML subsequent to confirmed CR after consolidation chemotherapy either underwent allogeneic hematopoietic stem cell transplantation, various non-curative antileukemic treatments, or best supportive care.

Patient data and consent to anonymized publication were provided after approval by the local ethics committee (ref. nr. UCT-42–2021) according to the 2013 Declaration of Helsinki. Patients were identified from the clinical cancer registry of the university cancer center and annotated based on manual chart review and archived medical records. Results from all inhouse bone marrow aspirations for this patient cohort were retrieved from the medical records and manually annotated.

### Cytomorphology

Bone marrow smears obtained from the posterior superior iliac spine with a dedicated, single-use bone marrow aspiration needle were prepared immediately after aspiration using the crush film technique [17] and were subsequently air-dried and stained with May-Grünwald-Giemsa staining in accordance with the ICSH guidelines [18]. Only bone marrow aspirates containing particles were analyzed. Slides were visualized with a Zeiss Axioskop 2 plus light microscope using a 63 × Zeiss oil immersion objective. Hematologic relapse was defined as bone marrow blasts ≥ 5% or reappearance of blasts in the blood [10]. Cytomorphological assessment for relapse was carried out in-house independently by two experienced investigators, an experienced technician, and a senior attending physician of the Department of Hematology/Oncology both with many years of cytomorphology experience. The in-house cytomorphology laboratory is certified by the German national accreditation body according to international standards and rules (Deutsche Akkreditierungsstelle, DAkkS) and regularly participates in internal and external quality assurance controls.

### Statistical analysis

R 4.0.3 [19] and ggplot2 3.3.2 [20] were used for statistical analyses, data reporting, and plotting. Comparative analyses for differences in proportion and other numerical variables between groups were performed using chi^2^ test and Mann–Whitney *U* test. The Kaplan–Meier method was used for estimation of the disease-free survival. Survival between patient groups was compared with the log-rank test. The reverse-KM method was used to estimate median follow-up time. Hazard rate of relapse was estimated by fitting a parametric exponential function via logistic regression and confidence intervals were calculated by case-base sampling (R’s casebase 0.10.1). A *P* value < 0.05 was considered statistically significant.

## Results

### Baseline characteristics of AML patients

**Fig. 1:**
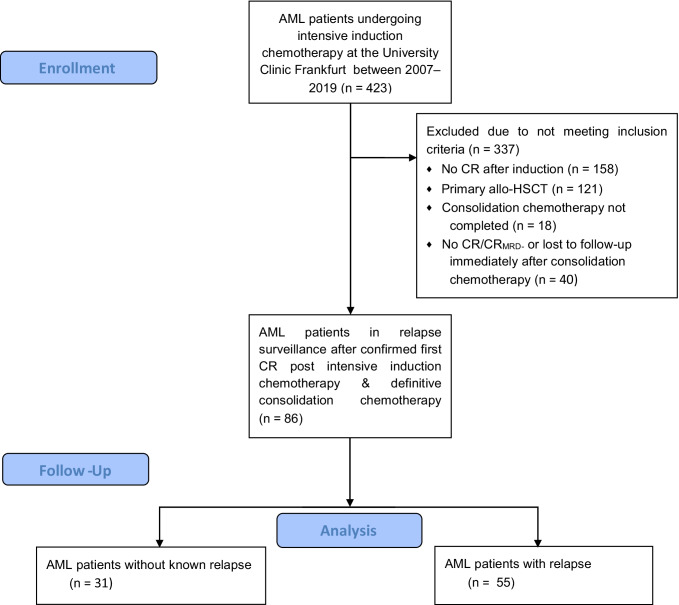
Study flow diagram

**Fig. 2:**
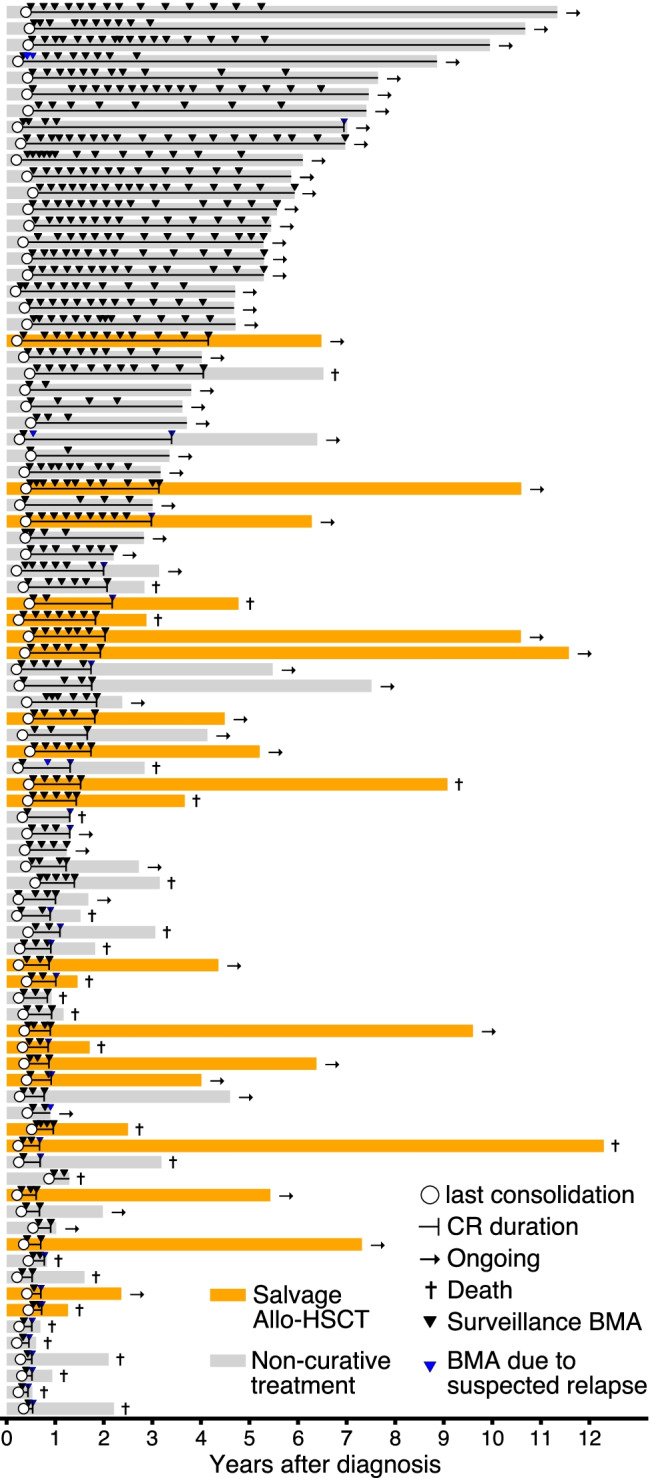
Individual patients, bone marrow aspirations, relapses, and outcome status. Swimmer plot showing individual patients from time of first diagnosis to death or last follow-up (*n* = 86). Each bar represents a patient. Bars are ordered by disease-free survival (complete remission (CR) duration) and color-coded by type of relapse therapy (allo-HSCT vs non-curative treatment). If relapse occurred, CR duration is marked by vertical line within each patient’s bar. All bone marrow aspirations for each patient between completion of consolidation chemotherapy and relapse or last follow-up are depicted as arrows and color-coded by whether they were conducted as part of the relapse surveillance program or due to specific suspicion of relapse. Each patient’s status (ongoing or deceased) is indicated

We identified 86 patients with newly diagnosed non-promyelocytic AML who underwent intensive induction chemotherapy between 2007 and 2019, followed by consolidation chemotherapy and confirmed complete remission (CR) after completion of consolidation chemotherapy (Fig. 1). A total of 55 AML patients (64%) were diagnosed with a later relapse of AML (Fig. 2). Descriptive statistics of the study group are shown in Table 1. Median age was 64 years (range, 21–78 years) in AML patients with relapse and 53 years (range, 26—74 years) in AML patients without relapse (*P* = 0.002). AML patients with relapse trended towards more adverse AML risk groups according to the European Leukemia Net (ELN) recommendations from 2010 [14]. Median follow-up time was 64 months (95% confidence interval (CI), 55–73 months).

**Table 1:** Baseline characteristics

		All patients (*n* = 86)	Relapse (*n* = 55)	No relapse (*n* = 31)	*P* value*
Gender	Female	46 (53%)	31 (56%)	15 (48%)	.63
	Male	40 (47%)	24 (44%)	16 (52%)	
Age at diagnosis, median (range), years		58 (21–78)	64 (21–78)	53 (26–74)	.002
WHO classification	AML with recurrent genetic abnormalities	50 (58%)	29 (53%)	21 (68%)	.08
	AML with dysplasia-related changes	2 (2%)	0 (0%)	2 (6%)	.26
	Therapy-related AML	1 (1%)	1 (2%)	0 (0%)	1
	AML, not otherwise specified	32 (37%)	25 (45%)	7 (23%)	.06
	Myeloid sarcoma	0 (0%)	0 (0%)	0 (0%)	
	Acute leukemias of ambiguous lineage	1 (1%)	0 (0%)	1 (3%)	.77
ELN2010	Favorable	30 (35%)	15 (27%)	15 (48%)	.08
	Intermediate-I	37 (43%)	25 (45%)	12 (39%)	.70
	Intermediate-II	16 (18%)	13 (24%)	3 (10%)	.19
	Adverse	3 (3%)	2 (4%)	1 (3%)	1
Bone marrow aspirations during remission, median (IQR)	All BMA	4 (3–9.8)	3 (3–5)	11 (4–14)	< .001
	Surveillance BMA	4 (2–9.5)	2.5 (1–4.3)	10 (3–13)	< .001
Relapse therapy	Allo-HSCT		23 (42%)		
	Palliative antileukemic therapy		28 (51%)		
	Death in relapse situation		2 (4%)		
	Unknown		2 (4%)		
DFS, median, months		19	8	(Not reached)	
OS, median, months		148	78	(Not reached)	

Count data is shown unless indicated otherwise. *IQR*, inter-quartile range; *ELN2010*, AML risk classification according to the European LeukemiaNet 2010 score [14]; *BMA*, bone marrow aspiration; *DFS*, disease-free survival; *OS*, overall survival

### Relapse surveillance and relapse therapy of AML patients in CR after consolidation chemotherapy

**Fig. 3:**
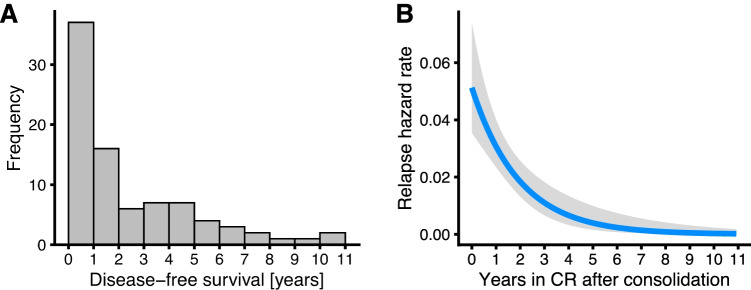
Relapses after consolidation chemotherapy with complete remission. **A** Empirical frequencies of disease-free survival times after consolidation chemotherapy with complete remission (CR). **B** Estimated hazard rate of relapse after consolidation chemotherapy with complete remission. Grey area shows 95% confidence interval of the estimated hazard rate

Relapse rates were 40%, 17%, 2%, 3%, and 0% during years 1 to 5, respectively. Disease-free survival times were distributed exponentially (Fig. 3A). The hazard rate of relapse was greatest in the first year after consolidation chemotherapy and decreased substantially over the subsequent years (Fig. 3B). AML patients with subsequent relapse (a competing risk for surveillance BMA) underwent a median of 3 (inter-quartile range (IQR), 3–5) surveillance BMAs during remission until relapse was diagnosed, whereas patients without relapse underwent a median of 11 (IQR, 4–14) surveillance BMAs (*P* < 0.001, Table 1). Relapse therapy consisted of allo-HSCT in 23 AML patients (42%) and palliative antileukemic therapy in 28 AML patients (51%, Table 1). Two AML patients (4%) died at the time of relapse and the remaining two AML patients (4%) were lost to follow-up after relapse diagnosis (Table 1).

### Diagnostic yield of relapse surveillance strategy by regular BMA

Our surveillance BMA regimen detected 29 out of 55 relapses (53%, Table 2). Eleven out of 55 relapses (20%) were diagnosed as molecular relapses based on newly onset MRD positivity by qPCR and 18 relapses (33%) were detected as morphological relapses (Table 2). The remaining 26 relapses (47%) were diagnosed as morphologic relapses by non-surveillance BMA prompted by specific suspicion of relapse. Two of these patients had persistence of dysplasia after consolidation therapy and relapsed with circulating myeloid blasts in peripheral blood between scheduled surveillance BMA. Among the 18 morphological relapses detected by surveillance BMA, the majority of AML patients (83%, *n* = 15) showed simultaneous abnormalities (circulating myeloid blasts and/or new thrombocytopenia/neutropenia/anemia) in PB at time of relapse and an additional 6% (*n* = 1) developed PB abnormalities within 2 weeks. In total, 7% (*n* = 3) of all morphological relapses were diagnosed by surveillance BMA in the absence of simultaneous overt PB abnormalities. Five percent (*n* = 2) of all morphological relapses continued to show no overt abnormalities in PB within 2 weeks of relapse diagnosis by BMA. Circulating blasts and thrombocytopenia were the most frequent diagnostic changes in PB upon relapse.

**Table 2:** Details on relapses

					DFS
All relapses				55 (100%)	
			Year 1	34 (62%)	
			Year 2	15 (27%)	
			Year 3	2 (4%)	
			Year 4	3 (5%)	
			Year 5	0 (0%)	
			Later	1 (2%)	
Detected by surveillance BMA	Yes			29 (53%)	10 (7–17)
		Molecular relapse		11 (20%)	
		Hematologic relapse		18 (33%)	
			BM blast percentage, median (IQR)	24 (9–44)	
			PB with peripheral blasts	13 (24%)	
			PB with lineage counts suspicious of relapse	13 (24%)	
			PB normal	3 (5%)	
			PB remaining normal within 2 weeks of BMA	2 (4%)	
	No			26 (47%)	7 (4–13)
		Hematologic relapse		26 (47%)	
			BM blast percentage, median (IQR)	27 (9–46)	
			PB with peripheral blasts	19 (35%)	
			PB with lineage counts suspicious of relapse	25 (45%)	
			PB normal	0 (0%)	

Count data is shown unless indicated otherwise. *DFS*, disease-free survival in months (95% confidence interval); *BMA*, bone marrow aspiration; *BM*, bone marrow; *PB*, peripheral blood

AML patients with relapse detected by surveillance BMA had a median disease-free survival of 10 months (95% CI, 7–17 months) compared to 7 months (95% CI, 4–13 months) in AML patients with relapse not detected by the surveillance program (*P* = 0.45, Table 2), although our study design does not permit a causal attribution of this difference to the surveillance regimen (see “Discussion” section). Individual AML patients, their clinical course, surveillance, and non-surveillance BMAs are shown in Fig. 2.

## Discussion

Current international and national guidelines recommend PB assessment every 1–3 months for 2 years and every 3–6 months for up to 5 years, and BMA only if PB smear is abnormal or cytopenia develop [2, 7, 8]. However, recommendations differ on how to extend this morphological relapse surveillance strategy to include molecular relapse surveillance [2, 7, 10, 21]. Furthermore, this surveillance strategy has never been specifically tested and compared to alternative surveillance strategies. Our single-center retrospective study evaluates the diagnostic utility of an intensive relapse surveillance strategy by additional (i.e., in addition to the consensus guideline recommendations) quarterly BMA in AML patients in first CR after consolidation chemotherapy for 2 years, followed by biannual BMA for up to 5 years.

Eighty-nine percent of relapses in our study population occurred within 2 years after consolidation chemotherapy, which is similar to data from others [22, 23]. Thus, AML patients are at far lower risk of relapse beyond 2 years after reaching consolidation therapy, suggesting a decreasing value of continued intensive relapse surveillance by BMA beyond this point.

Given typical AML proliferation kinetics, a sizeable percentage of relapses were diagnosed by the surveillance BMAs: in total, 53% of all relapses were detected by surveillance BMA (33% with morphological relapse, and additional 20% of relapses were diagnosed as molecular relapses). Seven percent of morphological relapses were only diagnosed by BMA without any parallel abnormalities in PB. However, it is probably not feasible to further increase the frequency of scheduled BMA. Thus, alternative strategies are required to increase the diagnostic yield. Several studies indicate that PB MRD monitoring by real-time qPCR is at least non-inferior to MRD assessment using bone marrow aspirates [21]. Furthermore, the majority of morphological relapses in our study demonstrated concurrent PB abnormalities and could thus have been diagnosed by PB monitoring only. An earlier study [4] analyzed paired bone marrow and peripheral blood evaluations in AML patients with relapse occurrence between 1980 and 1995 after CR and found that 16% of relapse diagnoses would have been delayed without bone marrow evaluation. The remaining majority of relapsing AML patients exhibited diagnostic PB abnormalities. Although upfront treatment regimens and stratification strategies were not reported and no specific surveillance strategy was implemented, this agrees remarkably well with our study showing that only 7% of morphological relapse diagnoses would have been delayed without BMA. Unfortunately, we lack the required data to determine exactly by how much time the relapse diagnosis would have been delayed for the minority of patients with no apparent PB abnormalities. However, a subset of these patients (33%) went on to develop PB abnormalities within 2 weeks, and, given AML proliferation kinetics, we speculate that most patients with ≥ 5% blasts detectable via BMA would have manifested with circulating blasts within a timeframe measured in weeks. Thus, more frequent (e.g., monthly) PB relapse monitoring could replace repeat BMA in most AML patients, might diagnose some patients with relapses occurring between more sparsely scheduled repeat BMA, and the delay in relapse detection in the remaining patients would probably be short, although nevertheless potentially of clinical importance.

Our retrospective study has several limitations. Cytomorphological assessment relies on the expertise of individual investigators and this may limit the generalizability of our findings. Although the described intensive relapse surveillance strategy is the standard follow-up procedure at our institution and recommended to all AML patients in remission after definitive consolidation chemotherapy, not all patients adhered to it strictly (see Fig. 2). Our study thus represents a real-world description of the implementation of this relapse surveillance strategy outside of a clinical trials context. Marginally more relapses may have been diagnosed by surveillance BMAs if more intense efforts were made to uniformly adhere to the surveillance strategy; however, this would probably not influence the rate of simultaneous peripheral blood abnormalities. Additionally, some patients were lost to follow-up after consolidation therapy, which leads to a selection bias. Finally, our study does not determine whether AML patients amenable for MRD monitoring should be managed differently. Our study did not investigate the potential impact of MRD flow cytometry in either BMA/PB or molecular real-time qPCR MRD monitoring on PB, as this was not systematically performed in our study population. Early MRD detection, preferably from peripheral blood, prior to overt relapse may allow early salvage therapy and allo-HSCT with low disease burden or enable specific MRD + maintenance therapy strategies, highlighting the clinical need for additional MRD monitoring.

In summary, our results may support an intensive relapse surveillance strategy of repeat BMA every 3 months during the first 2 years of remission for AML patients at high risk of relapse and a high likelihood to receive salvage therapy including entering clinical trials. Our data suggests to increase the PB sampling frequency to monthly surveillance during the first 2 years, followed by prompt BMA when relapse is suspected. Our data provides an empirical basis to discuss intensive relapse surveillance in the context of available treatment options and individual patients’ preferences and may help in this decision-making process. Sensitive MRD assays on PB may ultimately replace bone marrow evaluation for relapse surveillance in most AML patients.
